# Oral cancer: a major and growing public health problem towards a national policy of prevention and early detection in Tunisia

**DOI:** 10.11604/pamj.2020.37.291.25448

**Published:** 2020-12-01

**Authors:** Dorsaf Touil, Lamia Oualha, Nabiha Douki

**Affiliations:** 1 University of Monastir, Dental Faculty of Monastir, Research Laboratory LR12ES11, Monastir, Tunisia

**Keywords:** Oral cancer, prevention, public health

## Abstract

Oral cancer is a growing public health problem, it is increasing worldwide since it is the sixth most common cancer overall. The highest rates of oral cancer occur in the most disadvantaged sections of the population. Early detection and screening have been shown to be effective in reducing mortality and morbidity caused by oral cancers. To ensure early detection of oral cancer measures such as mass screening or screening of high-risk group population; reducing the delay from patients´ side by creating awareness about signs and symptoms of oral cancer. Today, no national population-based screening programs for oral cancer have been implemented yet. The objectives of this project are to evaluate and educate Tunisian Dentists as well as health professionals to perform oral screening and early detection of suspicious oral lesions in order to reduce morbidity and mortality associated with oral cancer.

## Perspective

Oral cancer is a growing public health problem that is constantly increasing worldwide since it is the sixth most common cancer overall [1]. Data collected from worldwide databases, such as Survival Epidemiology and End Result (SEER), the National Center of Health Statistics (NCHS) and GLOBCAN (2012) IARC reported that in 2012, 369,200 new cases of oral cancer were reported worldwide, with two-thirds of the tumors diagnosed in developing countries [2]. These cancers occur predominantly as squamous cell carcinomas (90%), and are highly lethal, incapacitating and disfiguring [3]. Moreover, these carcinomas have one of the lowest 5 year survival rates of all cancers. When diagnosed, they are often in a late and advanced stage leading to a higher morbidity and mortality rate [4].

The highest rates of oral cancer occur in the most disadvantaged sections of the population. An inequitable geographical distribution has been observed due to the regional differences in prevalence of disease-specific risk factors, accessibility and availability of screening/diagnostic measures, socio-economic factors and demographic parameters of the population [5]. Multiple socio-demographic and habit related risk factors such as male gender, older people, poor education status and socio-economic class are involved. Smoked as well as non-smoked forms of tobacco, alcohol, human papilloma virus infection, oral sex and genetic susceptibility have also been attributed in the causation of oral cancer [2]. Early detection and screening have been shown to be effective in reducing the mortality and morbidity. The American Cancer Society has recommended incorporating visual inspection of the oral cavity as part of a periodic health examination in dentist´s or physician´s office [5]. Similarly, The British Dental Association has also advocated screening high-risk group of patients opportunistically for oral cancer, when they attend for routine examination [6].

A variety of diagnostic aids and adjunctive techniques are available to potentially assist in the screening of healthy patients for evidence of otherwise occult cancerous change and to assess the biologic potential of clinically abnormal mucosal lesions. Recent clinical diagnostic tools include tolonium chloride or toluidine blue dye, Oral CDX® brush biopsy kits, salivary diagnostics and lastly optical imaging systems [7, 8]. To ensure early detection of oral cancer measures such as mass screening or screening of high-risk group population; reducing the delay from patients´ side by creating awareness about signs and symptoms of oral cancer, involvement of community members; and reducing the delay from doctors´ side by training health professionals to have a high index of suspicion in high-risk groups and routine oral screening during health check-ups; should be strategically formulated and implemented [9-11].

**Project objectives:** today, no national population-based screening programs for oral cancer have been implemented In Tunisia yet. The aims of this public project are to: educate the oral health professionals to perform oral screening as part of any routine dental examination (training, post-graduation programs and focus groups); involve other health professionals in the screening and early detection of any suspecious lesions: general practitioners, Oto-rhino-laryngologist (ORL) as well as maxillo-facial surgeons; recruite volunteers from the health community and to enroll them in a national database for tobacco and oral cancer advocacy.

**Activities and work plan:** a national evaluation of the knowledge and the information of Tunisian dentists with regards to oral cancer, the screening tools and methods: to reach this objective a cross sectional descriptive survey of colleges of American pathologists (CAP) type (Knowledge, attitudes and practice) of a representative sample of general dentist practicing in the private as well as the hospital sectors, will be carried over a period of one year. A national evaluation of the knowledge of general practitioner physicians, ORL as well as geriatricians, regarding the risk factors and the screening tools of oral cancer: for that, a seconds CAP survey will be carried to a representative sample of these physicians over a period of one year. A comprehensive advocacy training program for a core group of oral health professionals will be performed, the objectives of this training is to educate them how to perform correct mouth screening and to use of early diagnosis tools. Volunteers can also be enrolled in this training program. Develop and conduit a national campaign with the collaboration of the Tunisian ministry of health and the Tunisian ministry of education, to raise public awareness of oral cancer and its link to tobacco use and heavy alcohol consumption by creating a national oral cancer awareness week.

**Methods and approach:** we used the Tunisian population-based cancer registries to help develop an enhanced descriptive updated epidemiology of oral cancer in Tunisia. We made use of existing databases to address questions about oral cancer care, consequences, and costs in Tunisia. Two types of C.A.P surveys will be conducted to evaluate the knowledge of both Tunisian oral health professionals and general physicians about oral cancer and screening tools. According to the results of the surveys, comprehensive advocacy training programs for a core group of oral health professionals will be programmed in association with the Tunisian health ministry and volunteers. National campaigns to raise public awareness of oral cancer and its link to tobacco use and heavy alcohol consumption will be programmed. We will involve mass media and social networks to diffuse the information.

**Impact of the project:** by focusing on two areas: primary prevention (reducing risk factors) and early detection, we aim to reduce morbidity and mortality associated with oral cancer. Comprehensive education of medical and dental practitioners in diagnosing and promptly managing early lesions could facilitate the multidisciplinary collaboration necessary to detect oral cancer in its earliest stages. Furthermore, because of the public's lack of knowledge about the risk factors for oral cancer and because this disease can often be detected in its early stages, the public's awareness of oral cancer including its risk factors, signs, and symptoms, will significantly increase the onset of the disease.
